# FGFR2–BRD4 Axis Regulates Transcriptional Networks of Histone 3 Modification and Synergy Between Its Inhibitors and PD-1/PD-L1 in a TNBC Mouse Model

**DOI:** 10.3389/fimmu.2022.861221

**Published:** 2022-04-25

**Authors:** Josh Haipeng Lei, Lei Zhang, Zhenyi Wang, Raoul Peltier, Yusheng Xie, Ganchao Chen, Shiqi Lin, Kai Miao, Chu-Xia Deng, Hongyan Sun

**Affiliations:** ^1^ Cancer Center, Faculty of Health Sciences, University of Macau, Macau, Macau SAR, China; ^2^ Ministry of Education (MOE) Frontier Science Centre for Precision Oncology, University of Macau, Taipa, Macau SAR, China; ^3^ Department of Chemistry, City University of Hong Kong, Kowloon, Hong Kong SAR, China; ^4^ Department of Vascular Surgery, The Affiliated Hospital of Southwest Medical University, Luzhou, China; ^5^ Hefei National Laboratory for Physical Sciences at the Microscale and School of Life Sciences, University of Science and Technology of China, Heifei, China; ^6^ Department of Pharmacology, School of Basic Medical Sciences, Shandong University, Jinan, China; ^7^ Key Laboratory of Biochip Technology, Biotech and Health Centre, City University of Hong Kong, Shenzhen Research Institute, Shenzhen, China

**Keywords:** epigenetic, BRD4, FGFR2, TNBC, posttranslational modifications, immunotherapy

## Abstract

Epigenetic reprogramming is an independent mode of gene expression that often involves changes in the transcription and chromatin structure due to tumor initiation and development. In this study, we developed a specifically modified peptide array and searched for a recognized epigenetic reader. Our results demonstrated that BRD4 is not only an acetylation reader but of propionylation as well. We also studied the quantitative binding affinities between modified peptides and epigenetic regulators by isothermal titration calorimetry (ITC). Furthermore, we introduced the Fgfr2-S252W transgenic mouse model to confirm that this acetylation is associated with the activation of c-Myc and drives tumor formation. Targeted disruption of BRD4 in Fgfr2-S252W mouse tumor cells also confirmed that BRD4 is a key regulator of histone 3 acetylation. Finally, we developed a tumor slice culture system and demonstrated the synergy between immune checkpoint blockade and targeted therapy in triple-negative breast cancer (TNBC). These data extend our understanding of epigenetic reprogramming and epigenetics-based therapies.

## Introduction

**Figure 1:**
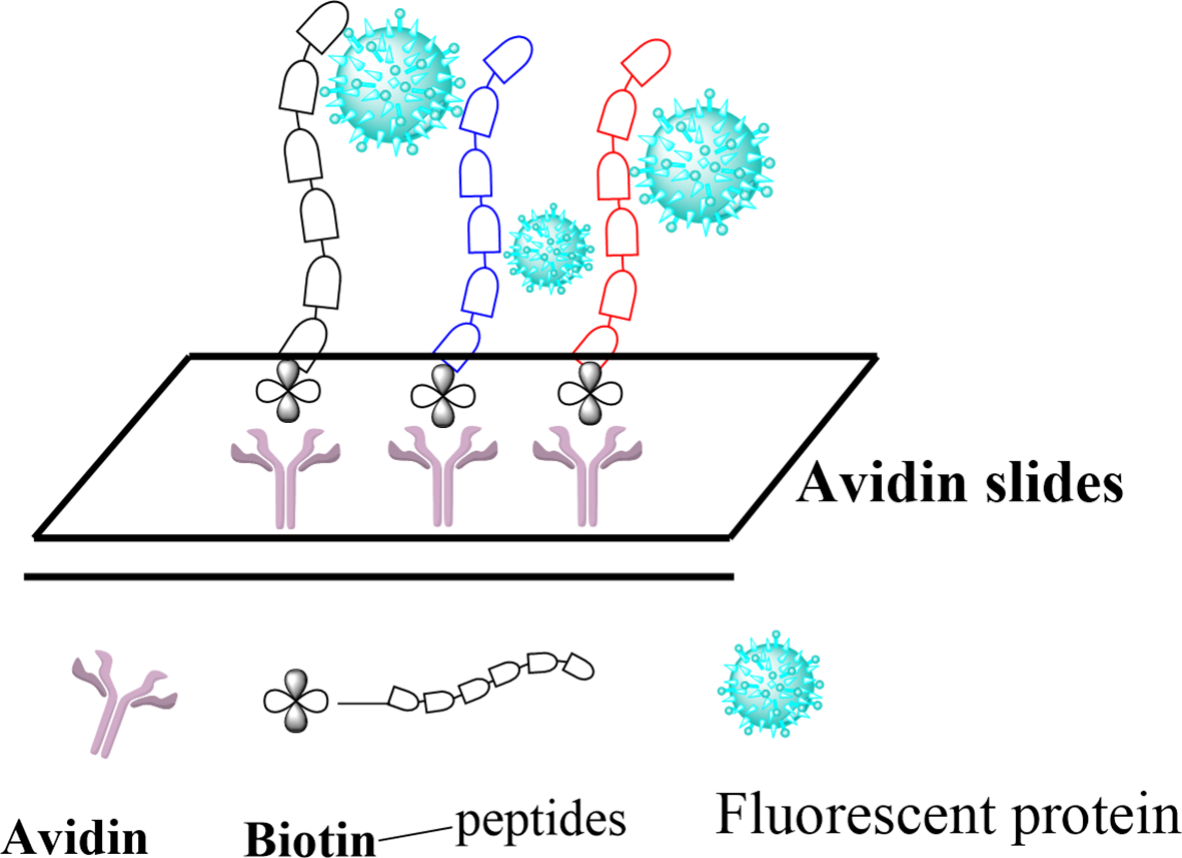
Overall screening workflow of the different acylated histone peptides by bromodomains.

Posttranslational modifications (PTMs) of histone proteins are key reactions in the regulation of the epigenetic machinery. They contribute to changing the structure and dynamics of chromatin and, hence, control gene transcription initiation and crucial events such as DNA replication, recombination, and repair (1–5). Among these, the acetylation of lysine residues is by far the most abundant and is known to be involved in regulating many important cellular functions, making the PTMs highly similar to protein phosphorylation regarding its prevalence and importance (6, 7). Other modifications typically include methylation (8), phosphorylation, ribosylation, biotinylation, citrullination, and SUMOylation (9–11), as well as the more recently discovered crotonylation, propionylation, butyrylation (12), and succinylation (13). Histone acetylation is also known to induce the recruitment of transcription factors and chromatin remodeling factors, leading to an enhanced transcriptional activity (14). These factors are usually recruited by an epigenetic reader domain in the proteins, known as bromodomain (BRD), which specifically recognizes *N*-acetylated lysine residues (15). Currently, BRDs are the only known interaction modules that specifically recognize the *N*-acetylation of lysine residues (16, 17). However, the ability of BRDs to recognize other modifications of lysine residues, especially those discovered most recently, has not been thoroughly studied so far. Flynn et al. reported on the ability of certain human BRDs to recognize the butyrylation and crotonylation modifications of histones (18). Here, we intend to further explore the affinity of BRDs to the recently discovered PTMs of lysine in histones by using peptide arrays as a tool to study peptide–protein interactions. We explore five modifications of histone 3 (H3) proteins, namely, acetylation, propionylation, butyrylation, crotonylation, and succinylation, at two different lysine positions (H3K9 and H3K56) and report on their recognition by two bromodomains: BRD4 (1) and BRD4 (2). Our approach, depicted in **Figure 1**, consists in immobilizing previously modified peptide sequences on a glass surface, incubating them with fluorescently labeled BRD4 (1) and BRD4 (2), and profiling the binding affinity *via* fluorescence measurement. Finally, we validate these findings using the Fgfr2-S252W triple-negative breast cancer (TNBC) mouse model.

## Material and Methods

### Preparing Functionalization Avidin Glass Slides

Firstly, an amine slide was prepared. The glass slides were immersed in the silane solution for at least 1 h with constant stirring. Secondly, the amine slides were functionalized with carboxylic acid using succinic anhydride. Thirdly, the carboxylic-functionalized slides were modified by *N*-hydroxysuccinimide (NHS), which were called NHS slides. Finally, the NHS slides were reacted with avidin to obtain avidin slides.

### Peptide Synthesis and Identification

The Liberty CEM Peptide Synthesizer was introduced to synthesize a range of peptides. The peptides were purified *via* semi-preparative HPLC and their characteristics were identified using the Applied Biosystems 4800 Plus MALDI-TOF/TOF (matrix-assisted laser desorption/ionization–tandem time of flight) analyzer.

### Protein Expression

Histidine (6×)-tagged BRD4A-c002 (1) and BRD4A-c011 (2) bromodomain expression constructs were used in this study. All constructs were transformed into competent *Escherichia coli* BL21 cells. Protein expression was induced with 0.1 mM IPTG overnight at room temperature. Thereafter, purification of the protein was performed with Ni-NTA agarose beads (Qiagen, Hilden, Germany) according to the manufacturer’s protocols. The protein concentration was measured using the BCA Protein Assay Kit (#23225). The proteins were labeled with Cy3-NHS ester. The purity of the protein was determined with 12% SDS-PAGE gel, followed by both in-gel fluorescence scanning and Coomassie staining.

### Microarray Preparation

For the peptide immobilization experiment, the concentrations of peptides in the spotting buffer (phosphate-buffered saline/dimethyl sulfoxide, PBS/DMSO = 1:1) were prepared in 384 wells before the spotting process. Proteins were prepared in PBS buffer before the spotting process. The spotting process was performed using an ESI SMA arrayer (Ontario, Canada), followed by incubation in a humidity chamber with different periods. After the incubation process, the PBST buffer was used to wash the slices, rinsed with deionized water, and dried with nitrogen. The slides were finally scanned with a microarray scanner.

### Isothermal Titration Calorimetry Assay

The Malvern Panalytical Ltd. microcalorimeter was used to determine the binding affinity of BRD4 to different peptides. There are several steps to this assay, including protein and peptide preparation, instrument washing, sample loading, titration, and data analysis.

### Animal Experiments

All animal experiments were approved by the University of Macau Animal Ethics Committee (under the protocol UMARE-015-2019). The generation of Fgfr2-S252W mice was described in a previous work (19). Transgenic mice were continually checked to accelerate the growth of mammary tumors. Tumor samples were cut using sharp blades into small pieces of about 0.1–0.3 cm in diameter and processed three ways, as follows: 1) two larger pieces were fixed in 10% formalin and processed for histology and immunohistochemistry (IHC) staining with antibodies; 2) three pieces were immediately frozen in liquid nitrogen and used for subsequent DNA, RNA, and protein isolation; and 3) all the remaining pieces were placed in a freezing medium [10% DMSO/25% serum/65% Dulbecco’s modified Eagle’s medium (DMEM)], slowly frozen to −80°C, and transferred into liquid nitrogen a few days later. Our data indicated that the tumor tissues were well preserved under this condition and can be used for initiating tissue culture and xenograft tumors in nude mice at a later time. Primary FGFR2 tumor cells were derived from the Fgfr2-S252W mouse model using a standard procedure and maintained in F-medium.

### Generation of Lentivirus BRD4 and Infection of Fgfr2-S252W Tumor Cells

Unique sgBRD4 sequences were individually cloned into the lentil-CRISPR v2 vector (Addgene plasmid #52961) with a puromycin resistance marker, and are listed in **Table S3**. For BRD4 lentivirus production, 9µg DNA(plasmid: pCMVR8.2(addgene plasmid #12263): pMD2-VSVG =4: 3: 2) was used for transfection per 10-cm dish, and the virus was filtered with a 0.45-µm filter 48 h later. Then, Fgfr2-S252W mammary tumor cells were infected with the virus 24 h later. Infected cells were selected after 3 days with 4 µg/ml puromycin (Invitrogen, Carlsbad, CA, USA). After selection, the cells were switched to F-medium for the subsequent experiment.

### Tumor Slice Culture

A tumor slice culture method was performed in tumors developed from the Fgfr2-S252W mouse model, as previously described. In brief, the tumors were collected and extracted by punch biopsy. Thick tissue slices (250 μm) were obtained with a vibratome (Leica VT1200 S). Then, the tissues were placed on inserts with a culture medium. After 4–6 days, the tissues were stained with MTT and the slice viability was measured.

### Western Blot Analysis

Total proteins of tumors or cells were extracted using RIPA buffer with phosphatase and a protease inhibitor. Following a previously described protocol, immunoblotting was carried out using ChemiDocTM with corresponding antibodies. The antibodies used for IHC and Western blot are listed in **Supplementary Table S2**.

### Real-Time PCR

The total RNAs of tumors or cells were extracted using TRIzol^®^ Reagent, then reversed to complementary DNA (cDNA) (kit #205313; Qiagen). Then, the transcriptional levels of the target genes were examined with specific primers using the QuantStudio™ 7 Flex Real-Time PCR System (Thermo Fisher Scientific, Waltham, MA, USA). Gene-specific data were normalized to 18S expression. The primers are listed in **Table S3**.

### Statistical Analysis

All values were presented as the mean ± SEM of individual samples. The samples were analyzed using unpaired two‐tailed *t*‐tests. A *p* < 0.05 was considered statistically significant. All analyses were conducted in GraphPad Prism 7 (GraphPad Software, La Jolla, CA, USA).

## Results

### Synthesis and Preparation of Histone 3 Modification Peptides

**Figure 2:**
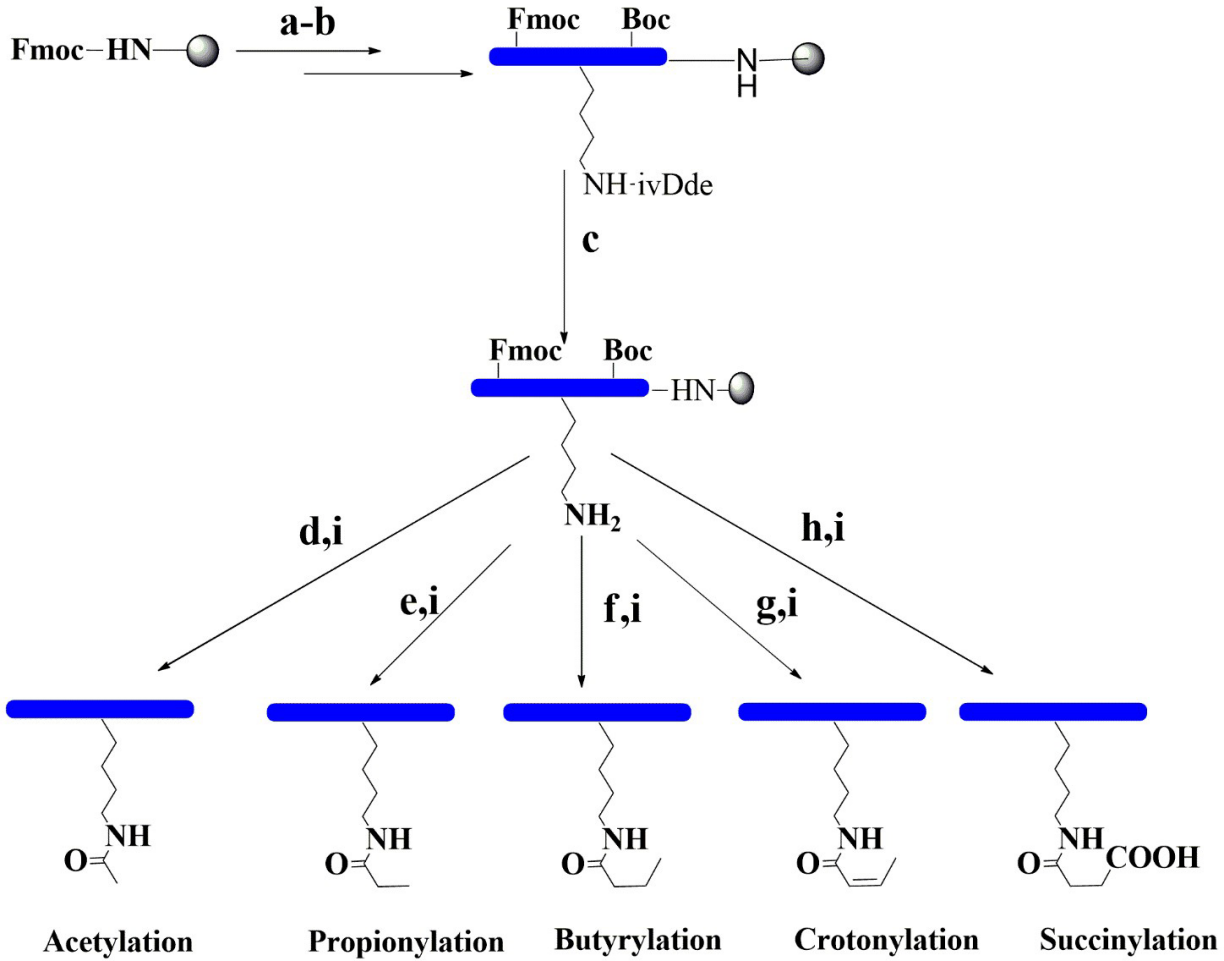
Synthetic pathways for the preparation of modification peptides. (a) With 20% piperidine/DMF. (b) Fmoc amino acid, HBTU, HOBT, and DIPEA. (c) With 4% hydrazine and DMF. (d) With 10% acetic anhydride and toluene. (e) With 10% propionic anhydride and toluene. (f) With 10% butyric anhydride and toluene. (g) With 10% crotonic anhydride and toluene. (h) With 10% succinic anhydride and DMF. (i) With 95% TFA, 2.5% TIS, and 2.5% H_2_O. *DIPEA*, *N*,*N*-diisopropylethylamine; *DMF*, *N*,*N*-dimethylformamide; *Fmoc*, fluorenylmethyl carbamate; *Boc*, *tert*-butyloxycarbonyl; *HOBT*, hydroxybenzotriazole; *HBTU*, *N*,*N*,*N*9,*N*9-tetramethyl-*O*-(1*H*-benzotriazol-1-yl)uronium hexafluorophosphate; *TFA*, trifluoroacetic acid; *TIS*, triisopropylsilane.

Firstly, the fully protected peptide sequences Biotin-GGIRRYQK(ivDde)STELL (H3K56) and Biotin-GGKQTARK(ivDde)STGGK (H3K9) were synthesized *via* conventional solid-phase chemistry using Fmoc-based synthesis (**Figure 2**). In both sequences, the ivDde group of lysine was then selectively removed on resin using 4% hydrazine in dimethylformamide (DMF). Subsequent modifications of the free lysine residue were carried out using 10% of the appropriate anhydride in toluene. The final biotin-containing peptides were then cleaved from the resin with concomitant removal of the protecting groups under optimized trifluoroacetic acid (TFA) cleavage conditions. In total, 12 modified peptides of H3 were synthesized in this study. Applied Biosystems 4800 Plus MALDI-TOF/TOF analysis was used to confirm the identity of the final peptides (**Supplementary Figure S1** and **Table S1**).

### Histone 3 Modification Peptide Recognition of the Human Bromodomain by Peptide Array

**Figure 3:**
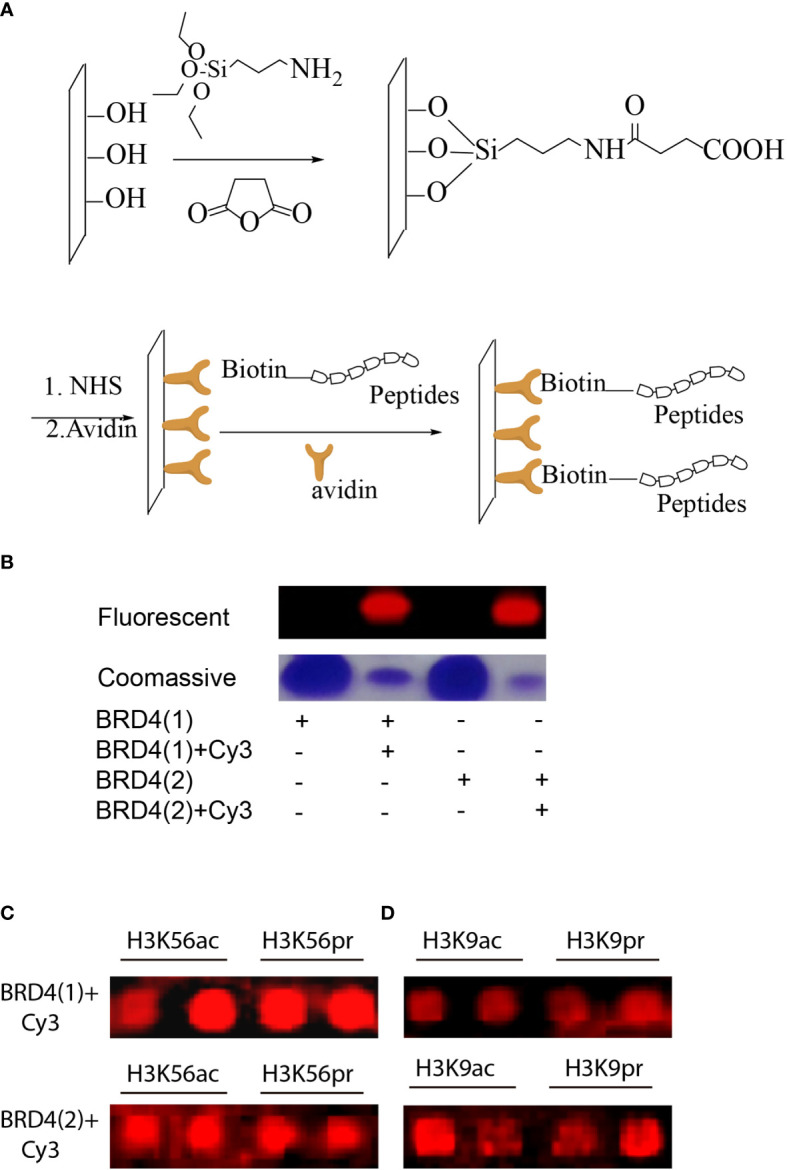
**(A)** Peptides with N-terminal biotin immobilized on avidin-functionalized slides. **(B)** Fluorescent and Coomassie gel images of representative fluorescent-labeled bromodomains (BRDs). **(C)** Microarray images of fluorescent-labeled BRD4 (1) binding to a small peptide library based on H3K56 or H3K9 modifications. **(D)** Microarray images of fluorescent-labeled BRD4 (2) binding to a small peptide library based on H3K56 or H3K9 modifications.

The peptides were then immobilized on a glass surface *via* the use of a highly chemoselective avidin–biotin ligation reaction. In this concept, an avidin-functionalized glass slide was quickly produced through some sequential chemical modification steps (**Figure 3A**). The peptides were then site-specifically immobilized onto the functionalized surface in 12 distinct subgrids. BRD4 (1) and BRD4 (2) were purified using Ni-NTA agarose beads (Qiagen) for purification (20). The proteins were labeled with Cy3-NHS and characterized using SDS-PAGE and Coomassie gel (**Figure 3B**). Each subgrid was then incubated with fluorescent-labeled BRD4 (1) and BRD4 (2) for half an hour. Then, the slide was washed copiously using 0.5% PBST buffer and scanned using a microarray scanner (**Figure 3C**). Based on the fluorescent intensity, we concluded that BRD4 could bind to various H3-modified peptides.

### Analysis of Histone3 Modification Peptide Binding Affinity to BRD4 by Isothermal Titration Calorimetry

**Figure 4:**
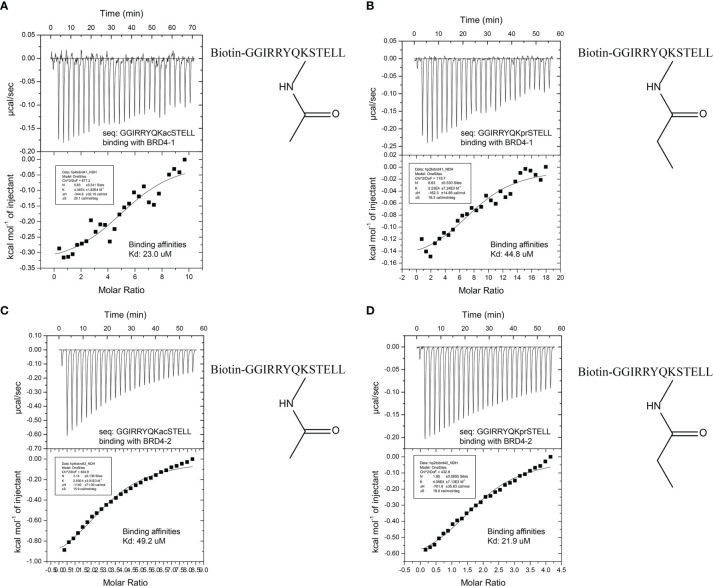
**(A)** Isothermal titration calorimetry (ITC) data of the acetylated histone peptide H3K56 (*Ac*) binding to BRD4 (1). **(B)** ITC data of the propionylated histone peptide H3K56 (*Pr*) binding to BRD4 (1). **(C)** ITC data of the acetylated histone peptide H3k56 (*Ac*) binding to BRD4 (2). **(D)** ITC data of the propionylated histone peptide H3k56 (*Pr*) binding to BRD4 (2).

The profiling experiment revealed several potent peptide binders for H3K56, with two peptide sequences, namely, GGIRRYQKacSTELL and GGIRRYQKprSTELL, showing particular affinity to both BRD4 (1) and BRD4 (2) (**Figures 3C, D**). A similar binding profile was observed with H3K9, but with fluorescence intensity lower than that for H3K56 (**Supplementary Figure S2**). In order to compare these results with better accuracy, the quantitative binding affinities of the H3K56 peptides in solution were measured by isothermal titration calorimetry (ITC). The ITC data for the two binding peptides, KAc- and KPr-modified H3K56, are shown in **Figure 4**. The dissociation constants (*K*_d_) for the acetylated peptide H3K56Ac were found to be 23.0 and 49.2 µM for binding to BRD4 (1) and BRD4 (2), respectively, which were similar to values previously reported in the literature (21). The propionylated sequence H3K56Pr showed an approximately 10-fold increase in binding affinity to BRD4 (1) and BRD4 (2) (*K*_d_ = 44.8 and 21.9 µM, respectively) compared with the previously reported *K*_d_ values for propionylated lysine peptides derived from histone H3 (13). Altogether, our peptide array experiments and ITC studies showed that BRD4 binds not only to *N*-acetylated lysine residues, as previously reported, but also to propionylated lysine residues. These results together demonstrated that peptide array technology is a powerful tool for studying peptide–protein interactions.

### BRD4 Regulates Histone 3 Modification in the Fgfr2-S252W Triple-Negative Breast Cancer Mouse Model

**Figure 5:**
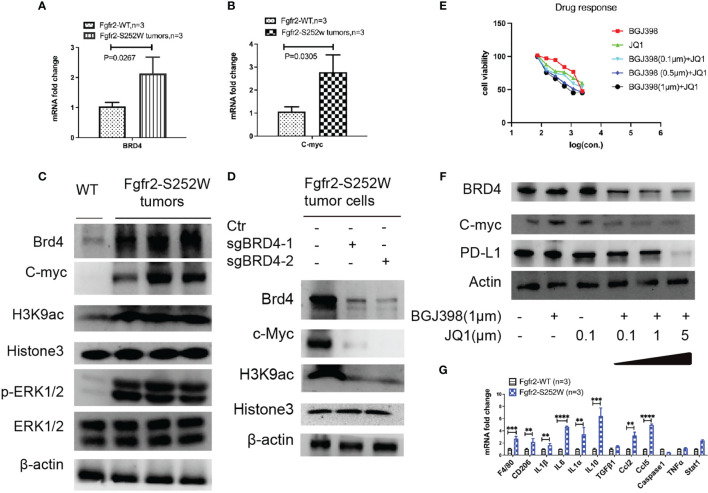
**(A, B)** RT-PCR analysis of the mRNA levels of Brd4 **(A)** and c-Myc **(B)** in tumors from the activation of Fgfr2-S252W transgenic mice. **(C)** Immunoblots with antibodies specific for the H3K9 acetylated lysine residues of histone H3 and pathway analysis of the activation of FGFR2 tumors or wild type (WT). **(D)** Immunoblots with antibodies specific for the H3K9 acetylated lysine residues of histone H3 and pathway analysis after knockout of BRD4 by the CRISPR-Cas9 system and non-target as the control. **(E)** Assessment of the drug response of BGJ398, JQ1, or the combined treatment of Fgfr2-S252W tumor cells with the Alamar blue assay. **(F)** Western blot analysis revealing that BGJ398 and JQ1 inhibit BRD4, c-Myc, and PD-L1. **(G)** Inflammation analysis using real-time RT-PCR from Fgfr2-S252W or WT mouse. P-values by using GraphPad Prism 7 Software. **p < 0.01, ***p < 0.001, ****p < 0.0001.

Recently, we have found that the activation of FGFR2 induces TNBC with multiple signaling, such as cancer stem cells (CSCs), epithelial–mesenchymal transition (EMT), and the tumor microenvironment (TME) (19). Thus, we examined whether BRD4 is involved in regulating the histone acetyltransferase (HAT) activity inactivation in the Fgfr2-S252W TNBC mouse model. We found that BRD4 was highly expressed in mouse tumors by multiple methods, such as RT-PCR (**Figure 5A**) and immunoblot (**Figure 5C**). Subsequently, we used specific H3K9ac and H3k56ac antibodies to examine whether BRD4 regulates the acetylation of the lysine residues in H3 in Fgfr2-S252W tumors. Our data showed that BRD4 was positively correlated with acetylated H3K9, and this acetylated residue was associated with open chromatin and active c-Myc (**Figures 5B, D** **)**. The knockout of BRD4 resulted in the suppression of H3K9ac acetylation activity (**Figure 5D**). Taking all these data together, we believe that FGFR2 activates the MAPK signaling pathway and regulates BRD4 and c-Myc expressions (**Figure 5C**). These data suggest that BRD4 is a potential target gene for cancer treatment. Afterward, we investigated whether BRD4 degradation is a targeted strategy for Fgfr-S252W mammary tumors. We obtained an Fgfr2-S252W cell line from the mouse model. The cells with Fgfr2-S252W were not very sensitive to the BRD4 inhibitor (BRD4i) JQ1 when compared to their response to the fibroblast growth receptor (FGFR) inhibitor (FGFRi) BGJ398 under the cell culture conditions (**Figure 5E**). Therefore, we combined FGFRi and BRD4i; the data showed that low concentrations of FGFRi (1 μM) and BRD4i (1 μM) had synergistic effects. However, Fgfr2-S252W tumor cells still showed resistance to BRD4i. Previous studies have shown that an elevated level of programmed death-ligand 1 (PD-L1) confers resistance to FGFRi. Thus, we next examined whether PD-L1 would induce BED4i resistance. We examined the levels of PD-L1 in various treatment groups (**Figure 5F**). We found that PD-L1 was significantly decreased only when FGFRi (BGJ398) was combined with a high concentration of JQ1 (5 μM). These findings suggest that PD-L1 promotes resistance to targeted therapy. Thus, we examined the expressions of inflammatory genes such as IL-1α, IL-1β, and IL-6. The data indicated that these pro-inflammatory mediators could induce the expressions of chemokines and the recruitment of macrophages (F4/80, CD206) to produce C–C motif chemokine ligand 2 (CCL2) or CCL5 (**Figure 5G**). Fgfr2 activation created an immunosuppressive environment and enhanced tumor progression. Thus, combination strategies using inhibitors targeting both FGF/FGFR and the bromodomain and extraterminal (BET) proteins with immune checkpoint blockade (ICB) may provide a useful approach for cancer treatment.

### Tumor Slice Culture System for the Synergy Between Immune Checkpoint Blockade and Targeted Therapy

**Figure 6:**
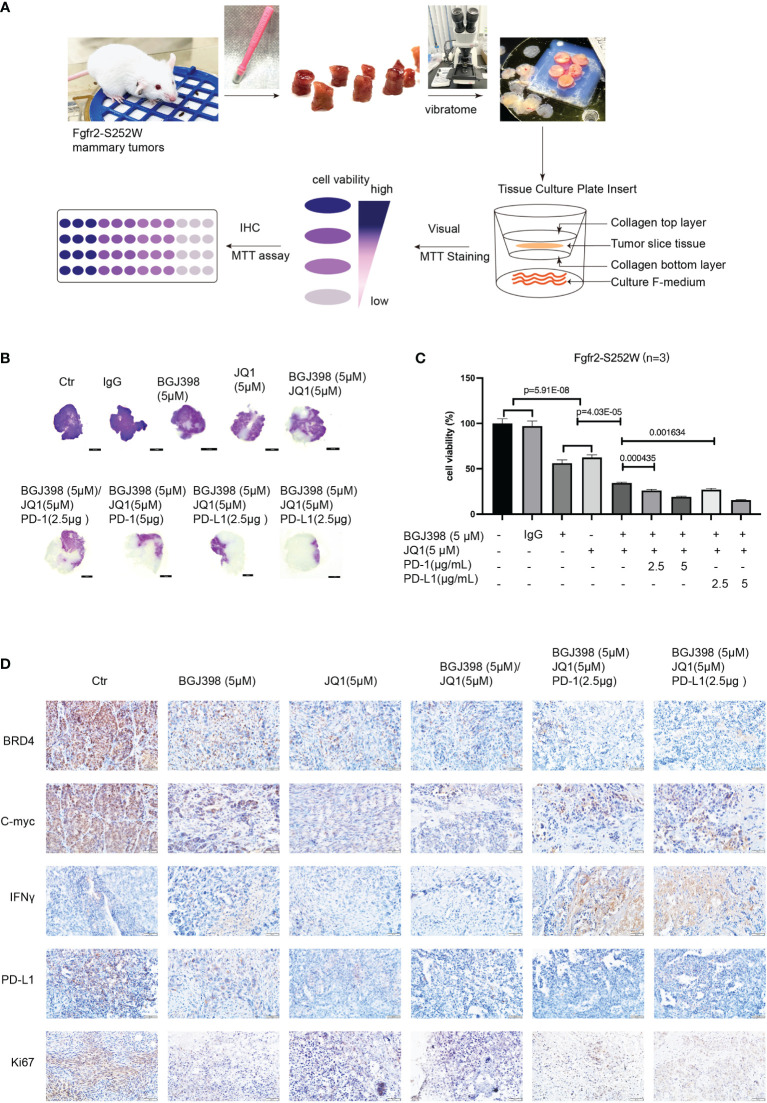
**(A)** Three-dimensional tissue slice culture workflow for quick, reliable models assessing immunotherapy. **(B)** Visualization of the antitumor response revealed by MTT analysis. **(C)** MTT assay evaluating the proliferation of Fgfr2-S252W tumor tissues. Data are shown as the mean ± SEM. p-Values by two-tailed Student’s t-test. **(D)** Immunohistochemistry (IHC) staining against BRD4, c-Myc, IFN-γ, Ki67, and PD-L1 in the indicated treatment groups.

It is important to establish quick and reliable models to assess targeted therapy or the combinations of immunotherapy for clinical therapeutics. To this end, we introduced the tumor slice culture platform to overcome PD-L1-mediated resistance due to this platform being able to maintain the activity of immune cells under the tissue culture condition (19). Firstly, equal-sized slices of mammary tumors were prepared from Fgfr2-S252W mice (**Figure 6A**) using a Biopunch (#15111-50). Thereafter, the slices were placed into a tissue slice culture insert with F-medium and treated with various drugs or in combination with ICB targeting tumor cells. The slices were then stained with MTT for 4–6 days after the culture. The data revealed that targeted therapy combined with ICB significantly increased the killing efficacy (**Figure 6B**). The data were also confirmed by colorimetric detection (**Figure 6C**). In addition, we performed IHC staining against BRD4, c-myc, IFN-γ, Ki67, and PD-L1 to explore the mechanisms underlying the synergistic actions between FGFR/BRD4 inhibitors and immunotherapy. As shown in **Figure 6D**, the levels of BRD4, c-Myc, PD-L1, and Ki67 significantly decreased, while the level of INF-γ markedly increased in the anti-programmed cell death 1 (PD-1)/PD-L1 group compared with the controls. Therefore, the tumor slice culture system can be used to quickly evaluate the efficacy and provide a versatile platform for immuno-oncology and drug discovery.

In summary, we have used a peptide array to demonstrate that BRD4 can recognize not only acetylated lysine residues of H3 but also propionylated lysine residues. We have successfully developed a fast and selective strategy to profile the binding affinity of lysine-modified peptides and proved that the immobilization method could serve as a powerful tool for mapping protein/substrate specificity. Furthermore, we validated this finding in the TNBC mouse model. The knockout study of BRD4 in Fgfr2-S252W mouse tumor cells suggested that BRD4 is a key regulator of H3K9 acetylation in TNBC. This acetylation is associated with the activation of c-Myc and drives tumor formation. Furthermore, we found that the combination of FGFR2i and BRD4i with ICB could significantly increase the sensitivity of cancer cells to immunotherapy.

## Discussion

Historically, the study of epigenetic development (22–24) included genomic methylation (both histone and DNA), histone modification, and loss of heterochromatin (25). The mechanisms by which these changes occur remain relatively unclear (26), although histone acetylation, methylation, phosphorylation, and ubiquitination have been studied and are well known (1, 2, 25). In general, histone PTMs are added using specific enzymes, which are categorized as writers. The writers include HATs, histone methyltransferases (HMTs)/histone lysine methyltransferases (KMTs), protein arginine methyltransferases (PRMTs), kinases, and ubiquitin ligases, among others. Readers are defined as the specialized domain-containing proteins which are capable of identifying and interpreting these modifications. Examples of readers include methyl CpG binding domains (MBDs), Tudor, plant homodomain (PHD), chromodomain, and bromodomain. Erasers belong to the group of enzymes that remove these chemical tags, including histone deacetylases (HDACs), histone demethylases (HDMs)/histone lysine demethylases (KDMs), phosphatases, and deubiquitinating enzymes (DUBs). In this study, we explored the readers, especially bromodomain (BRD4), which belongs to the BET family, and identified that it can read various acetylated lysine marks at H3 and H4 as well (17). Furthermore, in our study, we also found that BRD4 can recognize the propionyl group attached to a lysine amino acid residue of H3K9/56 by peptide array, which was further confirmed by ITC experiments. Importantly, we demonstrated that BRD4 is the key enzyme responsible for controlling the status of lysine acetylation at H3K9 in the Fgfr2-S252W TNBC mouse model. On the other hand, it has been reported (27) that BRD4 not only recognizes histone acetylation but also serves as a HAT. Our results suggest that histone modification in the TNBC mouse model may serve as a biomarker of cancer progression.

Recently, crosstalk of the BRD4/c-Myc axis in a TNBC subtype through integrin/FAK-dependent signaling has been reported (28). The BRD4/c-Myc axis also plays a significant role in the TME and in the maintenance of immunity; in addition, the expression of PD-L1 was suppressed by BRD4 inhibition in TNBC (29). Previously, we reported that the Fgfr2-S252W mouse model induced the high expression of PD-L1, suggesting that combining FGFR inhibitors (BGJ398 or AZD457) and a BRD4i (JQ1) may be beneficial for patients who show resistance to high PD-L1 expression. Besides, breast cancer 1 (BRCA1)-deficient cells were sensitized to the BET inhibitor, which reversed the MYC/TXNIP axis by inhibiting the activity of thioredoxin and elevating cellular oxidative stress, causing DNA damage that led to the death of BRCA1-deficient breast cancer cells (30, 31). On the other hand, Fgfr2-S252W also suppressed the expression of BRCA1 (19), which signified that BRD4 inhibition may be synthetically lethal with FGFR or PARP inhibitors through the induction of homologous recombination deficiency or the STAT3 and MAPK signaling pathways. In addition, several pathways are involved in cancer cell resistance to BET inhibitors, including the activation of receptor tyrosine kinases (RTKs), JAK-STAT, phosphatidylinositol 3-kinase (PI3K), AKT/mTOR, and MAPK/ERK pathways (32). In a previous study, we demonstrated that Fgfr2 activation could induce downstream pathways, including PI3K, MAPK, AKT, and STAT3. The activation of multiple pathways is always linked to drug resistance (33, 34). Given all the factors above, combination strategies using inhibitors targeting both FGF/FGFR and BET proteins with ICB may provide a useful approach for personalized therapy in clinical studies.

## Data Availability Statement

The data presented in the study are deposited in the PRIDE database repository, accession number PXD032850.

## Ethics Statement

The animal study was reviewed and approved by The University of Macau (UMARE-015-2019). Written informed consent was obtained from the owners for the inclusion of their animals in this study.

## Author Contributions

HS and C-XD designed and facilitated this study. JL, LZ, and ZW acquired the data. HS, C-XD, RP, and JL wrote the manuscript. JL, LZ, SL, YX, GC, RP, and KM conducted the experiments. All authors contributed to the article and approved the submitted version.

## Funding

This work was supported by the following grants: the Science Technology and Innovation Committee of Shenzhen Municipality (JCYJ20180507181654823), the National Natural Science Foundation of China (32122003, 21572190), the Science and Technology Development Fund, Macau SAR (no. FDCT-0048/2019/A1), and the Natural Science Foundation of China (NSFC) (82030094).

## Conflict of Interest

The authors declare that the research was conducted in the absence of any commercial or financial relationships that could be construed as a potential conflict of interest.

## Publisher’s Note

All claims expressed in this article are solely those of the authors and do not necessarily represent those of their affiliated organizations, or those of the publisher, the editors and the reviewers. Any product that may be evaluated in this article, or claim that may be made by its manufacturer, is not guaranteed or endorsed by the publisher.

## References

[1] Dawson MA Kouzarides T. Cancer Epigenetics: From Mechanism to Therapy. Cell (2012) 150:12–27. doi: 10.1016/j.cell.2012.06.013 22770212

[2] Graff J Tsai LH. Histone Acetylation: Molecular Mnemonics on the Chromatin. Nat Rev Neurosci (2013) 14:97–111. doi: 10.1038/nrn3427 23324667

[3] Kaelin WG Mcknight SL. Influence of Metabolism on Epigenetics and Disease. Cell (2013) 153:56–69. doi: 10.1016/j.cell.2013.03.004 23540690 PMC3775362

[4] Dancy BM Cole PA. Protein Lysine Acetylation by P300/CBP. Chem Rev (2015) 115:2419–52. doi: 10.1021/cr500452k PMC4378506 25594381

[5] Fu J Liao L Balaji KS Wei C Kim J Peng J. Epigenetic Modification and a Role for the E3 Ligase RNF40 in Cancer Development and Metastasis. Oncogene (2021) 40:465–74. doi: 10.1038/s41388-020-01556-w PMC7819849 33199825

[6] Jones PA Baylin SB. The Epigenomics of Cancer. Cell (2007) 128:683–92. doi: 10.1016/j.cell.2007.01.029 PMC3894624 17320506

[7] Lo PK Sukumar S. Epigenomics and Breast Cancer. Pharmacogenomics (2008) 9:1879–902. doi: 10.2217/14622416.9.12.1879 PMC2633440 19072646

[8] Toyota M Suzuki H Yamashita T Hirata K Imai K Tokino T. Cancer Epigenomics: Implications of DNA Methylation in Personalized Cancer Therapy. Cancer Sci (2009) 100:787–91. doi: 10.1111/j.1349-7006.2009.01095.x PMC11159488 19236379

[9] Bannister AJ Kouzarides T. Regulation of Chromatin by Histone Modifications. Cell Res (2011) 21:381–95. doi: 10.1038/cr.2011.22 PMC3193420 21321607

[10] Huang H Sabari BR Garcia BA Allis CD Zhao Y. SnapShot: Histone Modifications. Cell (2014) 159:458–458.e451. doi: 10.1016/j.cell.2014.09.037 25303536 PMC4324475

[11] Vaughan RM Kupai A Rothbart SB. Chromatin Regulation Through Ubiquitin and Ubiquitin-Like Histone Modifications. Trends Biochem Sci (2021) 46:258–69. doi: 10.1016/j.tibs.2020.11.005 PMC7954875 33308996

[12] Zhang K Chen Y Mang ZH Zhao YM. Identification and Verification of Lysine Propionylation and Butyrylation in Yeast Core Histones Using PTMap Software. J Proteome Res (2009) 8:900–6. doi: 10.1021/pr8005155 PMC2921183 19113941

[13] Vollmuth F Geyer M. Interaction of Propionylated and Butyrylated Histone H3 Lysine Marks With Brd4 Bromodomains. Angew Chem-Int Ed (2010) 49:6768–72. doi: 10.1002/anie.201002724 20715035

[14] Morgan M Shilatifard A. Reevaluating the Roles of Histone-Modifying Enzymes and Their Associated Chromatin Modifications in Transcriptional Regulation. Nat Genet (2020) 52:1271–81. doi: 10.1038/s41588-020-00736-4 33257899

[15] Filippakopoulos P Knapp S. The Bromodomain Interaction Module. FEBS Lett (2012) 586:2692–704. doi: 10.1016/j.febslet.2012.04.045 22710155

[16] Filippakopoulos P Knapp S. Targeting Bromodomains: Epigenetic Readers of Lysine Acetylation. Nat Rev Drug Discov (2014) 13:339–58. doi: 10.1038/nrd4286 24751816

[17] Ferri E Petosa C Mckenna CE. Bromodomains: Structure, Function and Pharmacology of Inhibition. Biochem Pharmacol (2015) 106:1–18. doi: 10.1016/j.bcp.2015.12.005 26707800

[18] Flynn EM Huang OW Poy F Oppikofer M Bellon SF Tang Y. A Subset of Human Bromodomains Recognizes Butyryllysine and Crotonyllysine Histone Peptide Modifications. Structure (2015) 23:1801–14. doi: 10.1016/j.str.2015.08.004 26365797

[19] Lei JH Lee MH Miao K Huang ZB Yao ZC Zhang AP. Activation of FGFR2 Signaling Suppresses BRCA1 and Drives Triple-Negative Mammary Tumorigenesis That Is Sensitive to Immunotherapy. Adv Sci (2021) 8:1–17. doi: 10.1002/advs.202100974 PMC8564435 34514747

[20] Zhang CJ Tan CYJ Ge JY Na ZK Chen GYJ Uttamchandani M. Preparation of Small-Molecule Microarrays by Trans-Cyclooctene Tetrazine Ligation and Their Application in the High-Throughput Screening of Protein-Protein Interaction Inhibitors of Bromodomains. Angew Chem-Int Ed (2013) 52:14060–4. doi: 10.1002/anie.201307803 24353229

[21] Filippakopoulos P Picaud S Mangos M Keates T Lambert JP Barsyte-Lovejoy D. Histone Recognition and Large-Scale Structural Analysis of the Human Bromodomain Family. Cell (2012) 149:214–31. doi: 10.1016/j.cell.2012.02.013 PMC3326523 22464331

[22] Fernandez-Barrena MG Arechederra M Colyn L Berasain C Avila MA. Epigenetics in Hepatocellular Carcinoma Development and Therapy: The Tip of the Iceberg. Jhep Rep (2020) 2:1–14. doi: 10.1016/j.jhepr.2020.100167 PMC7585149 33134907

[23] Henshall DC. Epigenetics and Noncoding RNA: Recent Developments and Future Therapeutic Opportunities. Eur J Paediatr Neurol (2020) 24:30–4. doi: 10.1016/j.ejpn.2019.06.002 31235424

[24] O'donnell KJ Meaney MJ. Epigenetics, Development, and Psychopathology. Annu Rev Clin Psychol (2020) 16:327–50. doi: 10.1146/annurev-clinpsy-050718-095530 32084320

[25] Alhamwe BA Khalaila R Wolf J Von Bulow V Harb H Alhamdan F. Histone Modifications and Their Role in Epigenetics of Atopy and Allergic Diseases. Allergy Asthma Clin Immunol (2018) 14:1–16. doi: 10.1186/s13223-018-0259-4 PMC5966915 29796022

[26] Hanahan D. Hallmarks of Cancer: New Dimensions. Cancer Discov (2022) 12:31–46. doi: 10.1158/2159-8290.CD-21-1059 35022204

[27] Devaiah BN Case-Borden C Gegonne A Hsu CH Chen QR Meerzaman D. BRD4 Is a Histone Acetyltransferase That Evicts Nucleosomes From Chromatin (Vol 23 2016). Nat Struct Mol Biol (2017) 24:194–4:pg 540. doi: 10.1038/nsmb0217-194c PMC4899182 27159561

[28] Zhang Y Xu BW Shi JF Li JM Lu XL Xu L. BRD4 Modulates Vulnerability of Triple-Negative Breast Cancer to Targeting of Integrin-Dependent Signaling Pathways. Cell Oncol (2020) 43:1049–66. doi: 10.1007/s13402-020-00537-1 PMC7716866 33006750

[29] Jing X Shao S Zhang YJ Luo AQ Zhao L Zhang LF. BRD4 Inhibition Suppresses PD-L1 Expression in Triple-Negative Breast Cancer. Exp Cell Res (2020) 392:1–10. doi: 10.1016/j.yexcr.2020.112034 32339606

[30] Sun CY Yin J Fang Y Chen J Jeong KJ Chen XH. BRD4 Inhibition Is Synthetic Lethal With PARP Inhibitors Through the Induction of Homologous Recombination Deficiency. Cancer Cell (2018) 33:401–16. doi: 10.1016/j.ccell.2018.01.019 PMC5944839 29533782

[31] Zhang BY Lyu JF Liu YF Wu CJ Yang EJ Pardeshi L. BRCA1 Deficiency Sensitizes Breast Cancer Cells to Bromodomain and Extra-Terminal Domain (BET) Inhibition. Oncogene (2018) 37:6341–56. doi: 10.1038/s41388-018-0408-8 30042414

[32] Shu S Wu HJ Ge JY Zeid R Harris IS Jovanovic B. Synthetic Lethal and Resistance Interactions With BET Bromodomain Inhibitors in Triple-Negative Breast Cancer. Mol Cell (2020) 78:1096–113.e1098. doi: 10.1016/j.molcel.2020.04.027 PMC7306005 32416067

[33] Nussinov R Tsai CJ Jang H. A New View of Pathway-Driven Drug Resistance in Tumor Proliferation. Trends Pharmacol Sci (2017) 38:427–37. doi: 10.1016/j.tips.2017.02.001 PMC5403593 28245913

[34] Liu R Chen YW Liu GZ Li CX Song YR Cao ZW. PI3K/AKT Pathway as a Key Link Modulates the Multidrug Resistance of Cancers. Cell Death Dis (2020) 11:1–12. doi: 10.1038/s41419-020-02998-6 PMC7515865 32973135

