# Supplementary material for: FGFR2–BRD4 Axis Regulates Transcriptional Networks of Histone 3 Modification and Synergy Between Its Inhibitors and PD-1/PD-L1 in a TNBC Mouse Model
Source: Front Immunol. 2022 Apr 25;13:861221. doi: 10.3389/fimmu.2022.861221 (PMC9084888; doi:10.3389/fimmu.2022.861221)
Supplement: Supplementary file 1 [file DataSheet_1.pdf]

frontiers

Supplementary Material

FGFR2-BRD4 axis regulates transcriptional networks of histone3 modification and synergy between its inhibitors and PD-1/PD-L1 in TNBC mouse model

Josh Haipeng Lei[1,2,3], Lei Zhang[1,5,] Zhenyi Wang[6], and Raoul Peltier [3], Yusheng Xie[3,7], Ganchao Chen[3], Siqi Lin[1,2], Kai Miao[1,2], Chu-Xia Deng[1,2]*, Hongyan Sun[3,4]*

1 Cancer Center, Faculty of Health Sciences, University of Macau, Macau Macau SAR, 999078 China

2 MOE Frontier Science Centre for Precision Oncology, University of Macau, Taipa, Macau Macau SAR, 999078 China

3 Department of Chemistry, City University of Hong Kong, 83 Tat Chee Avenue, Kowloon, Hong Kong, P. R. China

4 Key Laboratory of Biochip Technology, Biotech and Health Centre, City University of Hong Kong, Shenzhen Research Institute, Shenzhen 518057, China

5 Department of Vascular Surgery, The Affiliated Hospital of Southwest Medical University, Luzhou, Sichuan, 646000 China

6 Hefei National Laboratory for Physical Sciences at the Microscale and School of Life Sciences, University of Science and Technology of China, Heifei, Anhui, 230026, China

7 Department of Pharmacology, School of Basic Medical Sciences, Shandong University, Jinan 250012, China

* Corresponding Author
E-mail: hongysun@cityu.edu.hk; cxdeng@um.edu.mo

Keywords: Epigenetic, Posttranslational modifications, BRD4, FGFR2, TNBC.

Material and Methods

All chemicals and solvents were purchased from commercial suppliers and used without further purification. Mass spectra (MS) were obtained on an applied Biosystems 4800 plus MALDI-TOF/TOF (Matrix-assisted laser desorption/ionization time-of flight) analyzer. HPLC purification was carried out on a 1525 Waters HPLC system equipped with a 2489 UV/Visible Detector.

1.2 **Amine slides:**

For this step use two specific “squarish” glass jar which are in the drawer labeled “glassware for silane treatment”. Pour 380 mL of 100 % ethanol, add 8 mL of water and 12 mL of

aminopropyltriethoxisilane. Add a stir bar (add the small stir bar otherwise it's too big and can't fit below the slide tray!), and stir for 10 minutes, with the glass cover on top. In the meantime, transfer you slides, freshly dried after the piranha treatment, onto a metal tray. Then soak the tray in the slide jar for at least one hour (2 hours is better but don't leave them longer or the silane solution will turn white and your slides will be spoilt) with constant stirring (and with the glass lid!).

Afterwards, take a green plastic jar (located in the same drawer "slide silanization") and fill it with 95% ethanol and soak-wash your slides about 3 times. Then place the whole tray in a glass slide dish (with the lid) and place everything in the 150 °C oven. Make sure that the slides are not stuck to each other, and to make sure of that try to keep the slide tray slightly "slanted". Similarly, in the 150 °C oven, put the petri dish with the slides inclined to avoid the slides sticking to each other. Cure for at least 2 hours, but you can also leave them overnight. Then, take them out, let it cool down and wash them with 95 % ethanol a few times (ethanol wash bottle) and dry as described above.

The slides can be stored in slide box for long period of times until you need them.

Silanes are kept in the 4 °C fridge, the 150 °C oven is in the core facility room. These slides can be stored in slide box for long period of times until you need them.

1.3 Glass slide functionalization with CO_2H

Place the amine slides in a metal slide tray. Use a slide dish and fill it with about 650 mL of DMF. Add 12 g of succinic anhydride (with a stir bar!), and when it's completely dissolved add 30 mL of 1 M $Na_2B_4O_7$ pH 9, and subsequently place the slide tray to soak, you can hang it on the stand in the fume hood. React it for 15-20 minutes. Shake the slide tray once in a while to avoid small air bubbles on the slides. In the meantime, on the 2nd hot plate start boiling deionized water (with stir bar too), until it reaches about 95 °C. After 20 minutes, take the slides out of the 1rst dish and soak them for 2 minutes in boiling water with constant stirring, make sure you keep them immersed in the water at all times. Then take the slides out and rinse copiously with ethanol (wash bottle). Dry briefly with nitrogen and finish drying in the fume hood.

The slides can be stored in slide boxes for long period of times.

Succinic anhydride is with common chemicals on the shelves.

These slides can be stored in slide box for long period of times until you need them.

1.4 Glass slide functionalization with NHS

Place the carboxylic functionalized slides in the boxes with red label (bottom is filled with DMF) on the slide bench. Prepare 12 mL of a solution of TBTU, DIEA and NHS (100 mM: 200mM: 100mM) in DMF, i.e. 12 mL DMF, 384 mg TBTU, 138 mg NHS, and 440 µL of DIEA, and pour the solution on top of the CO_2H slides. 12 mL is fine for about 30 slides (corresponds to 7 boxes with 4 slides each). Place the big cover slips (20 × 60 mm) on the slides, close the boxes and react for 3 hours. Afterwards, transfer the slides to a metallic tray. Wash the slides copiously with 95 % ethanol, using the wash bottles, holding the tray and flushing ethanol, these steps takes about 3-5 minutes. Don't wash the NHS slides for a longer period of time.

Alternatively, you can do this step in the slide jar by preparing about 500 mL of the same solution, but this uses up a lot of NHS so it's actually better to do this step with the slide boxes. Be careful, store these slides in the dessicator under nitrogen (one located below Mahesh's bench, the other one below Marie's bench).

▯ TBTU and NHS are either in – 20 °C freezer of with peptide synthesis chemicals (→ Mahesh). DIEA is below the fume hood. The big cover slips (20 × 60 mm) are located with the slide glassware. These slides should be stored in a dessicator under nitrogen (in the lab, there two of them, one located below Mahesh's bench, the other one below Marie's bench). So far, we've stored the NHS slides in dessicators at room temperature, but it could be advisable to put the dessicator in the cold room. However, in this case, make sure this does not result in condensation on the slides when you take them out of the cold room.

1.5 Glass slide functionalization with avidin

Place the epoxy slides on the boxes on the slide bench (boxes with blue label) and prepare a solution of of 1 mg/mL avidin in 10 mM $NaHCO_3$. Pour about 40 –60 µL of this solution onto the slides and 3 place a cover slip (use small or big cover slips depending on the area you want to functionalize) and incubate for 30 minutes. Then transfer the slides to a slide tray and wash with deionised water. Dry briefly with nitrogen and finish drying in the fume hood. Then place the slides back on the slides boxes and quench the remaining epoxide with a solution of 2 mM aspartic acid in a 0.5 M $NaHCO_3$ buffer (pH 9). Transfer the slides to a slide tray, wash with water, dry briefly with nitrogen and finish drying in the fume hood. Usually, avidin slides were prepared just when needed but since avidin is stable "as a rock", they should be OK if stored in the fridge.

Supplementary Figures and Tables

Table.1 Peptide Sequences

Peptides	Sequence
H3K56	Biotin-GGIRRYQ*K*STELL
H3K56(Ac)	Biotin-GGIRRYQ*K(Ac)*STELL
H3K56(Pr)	Biotin-GGIRRYQ*K(Pr)*STELL
H3K56(Bu）	Biotin-GGIRRYQ*K(Bu)*STELL
H3K56(Cr)	Biotin-GGIRRYQ*K(Cr)*STELL
H3K56(Su)	Biotin-GGIRRYQ*K(Su)*STELL
H3k9	Biotin-GGKQTAR*K*STGGK
H3k9(Ac)	Biotin-GGKQTAR*K(ac)*STGGK
H3k9(Pr)	Biotin-GGKQTAR*K(pr)*STGGK
H3k9(Bu)	Biotin-GGKQTAR*K(Bu)*STGGK
H3k9(Su)	Biotin-GGKQTAR*K(Su)*STGGK

Table 2 Primary antibody

Primary antibody	Brand	Model / Cat. #	Rate
Phospho-p44/42 MAPK (Erk1/2)	cst	#4370L	1:1000
p44/42 MAPK (Erk1/2)	cst	#4695S	1:1000
PD-L1 (E1L3N®) XP®	cst	#13684	1:1000
BRD4 Antibody	bethyl	A301-985A100	1:500(WB)<br>1:100(IHC)
c-Myc (D84C12) Rabbit mAb	cst	#5605	1:1000
Acetyl-Histone H3 (Lys9) (C5B11)	cst	#9649	1:1000
Acetyl-Histone H3 (Lys56)	cst	#4243	1:1000
Histone H3 (D1H2)	cst	#4499	1:1000
β-Actin (8H10D10)	cst	#3700	1:1000

Table 3. Oligonucleotides and Primers

	sgRNA
sg-mBrd4-F1	GGGCCTGCGTTGTAGACATT
sg-mBrd4-R1	aaacAATGTCTACAACGCAGGCCC
sg-mBrd4-F2	GCTGGAAAGGCCACGCAAAC
sg-mBrd4-R2	aaacGTTTGCGTGGCCTTTCCAGC
	Primers for Real-Time PCR (5'-3')
Q-mBRD4-F	GTGAGAAGCTAGGCCGTGTAG
Q-mBRD4-R	AGGCAGGACCTGTTTCAGAGT
Q-mC-myc-F	CCCAGCGAACGTGATGATAAT
Q-mC-myc-R	GCTTCCTCGGTTGGATATAGGAT
Q-mIL-1α-F1	ACGTCAAGCAACGGGAAGAT
Q-mIL-1α-R1	AAGGTGCTGATCTGGGTTGG

Q-mCCL2 -F1	ATTCTGTGACCATCCCCTCAT
Q-mCCL2 -R1	TGTATGTGCCTCTGAACCCAC
Q-mTGFβ1 -F1	AAGTTGGCATGGTAGCCCTT
Q-mTGFβ1 -R1	GCCCTGGATACCAACTATTGC
Q-mSTAT1 -F1	CTGAATATTTCCCTCCTGGG
Q-mSTAT1 -R1	TCCCGTACAGATGTCCATGAT
Q-mIL1β -F1	CTGGTGTGTGACGTTCCCATTA
Q-mIL1β -R	CCGACAGCACGAGGCTTT
Q-mCaspase-1 -F1	GCCCACTGCTGATAGGGTGA
Q-mCaspase-1 -R1	CCCGGGAAGAGGTAGAAACG
Q-mCcl5-F1	GCTGCTTTGCCTACCTCTCC
Q-mCcl5-R1	TCGAGTGACAAACACGACTGC
Q-mCd206-F	CTCTGTTCAGCTATTGGACGC
Q-mCd206-R	CGGAATTTCTGGGATTCAGCTTC
Q-mF4/80-F	TGACTCACCTTGTGGTCCTAA
Q-mF4/80-R	CTTCCCAGAATCCAGTCTTTCC
Q-mIL-6-F	TGGGGCTCTTCAAAAGCTCC
Q-mIL-6-R	AGGAACTATCACCGGATCTTCAA
Q-mIL-10-F	CCCATTCCTCGTCACGATCTC
Q-mIL-10-R	TCAGACTGGTTTGGGATAGGTTT
18S-F	ACCGCAGCTAGGAATAATGGA
18S-R	GCCTCAGTTCCGAAAACCA

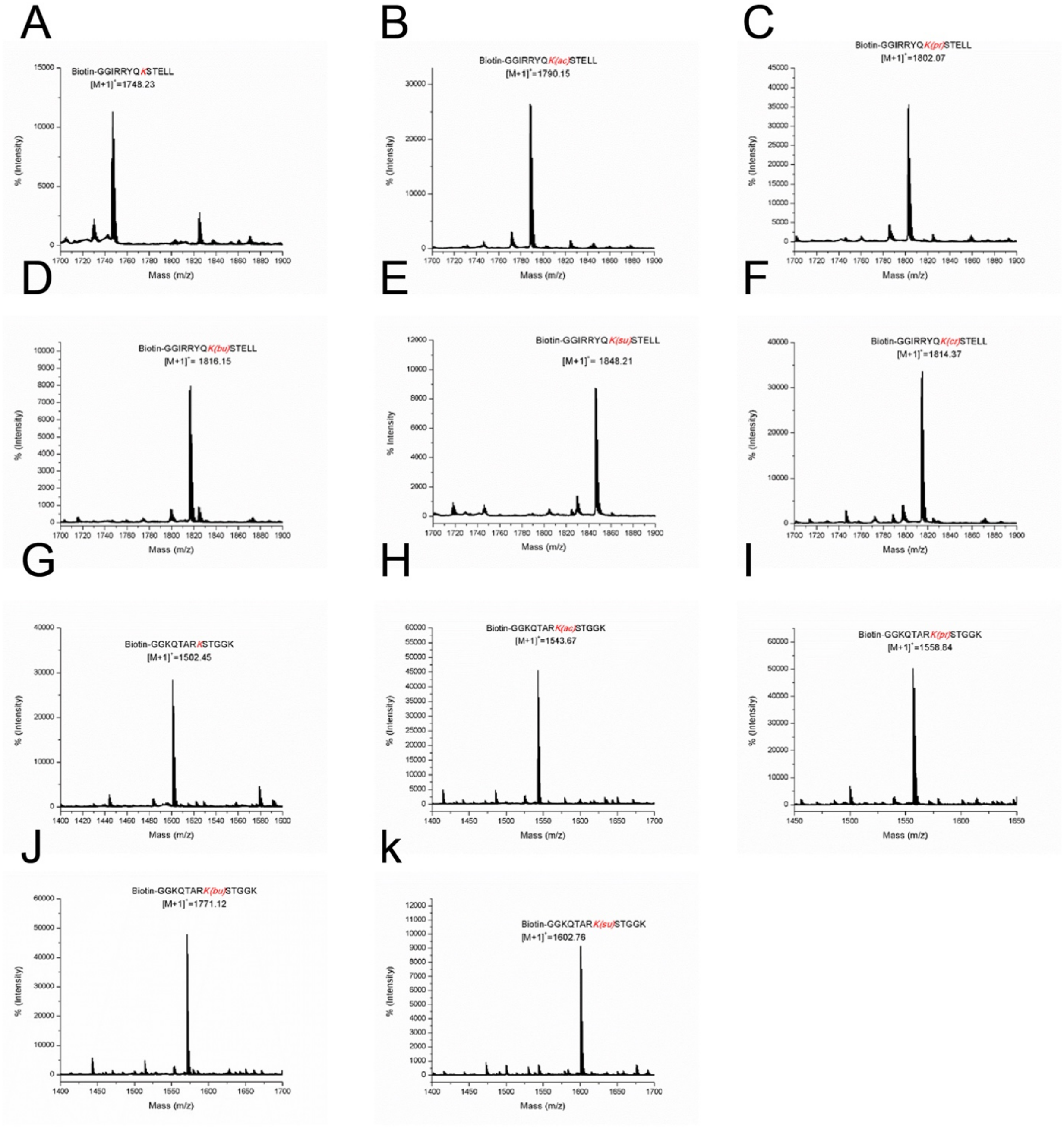

Figure S1 MALDI-TOF/TOF-MS/MS analysis of the 11 peptides.

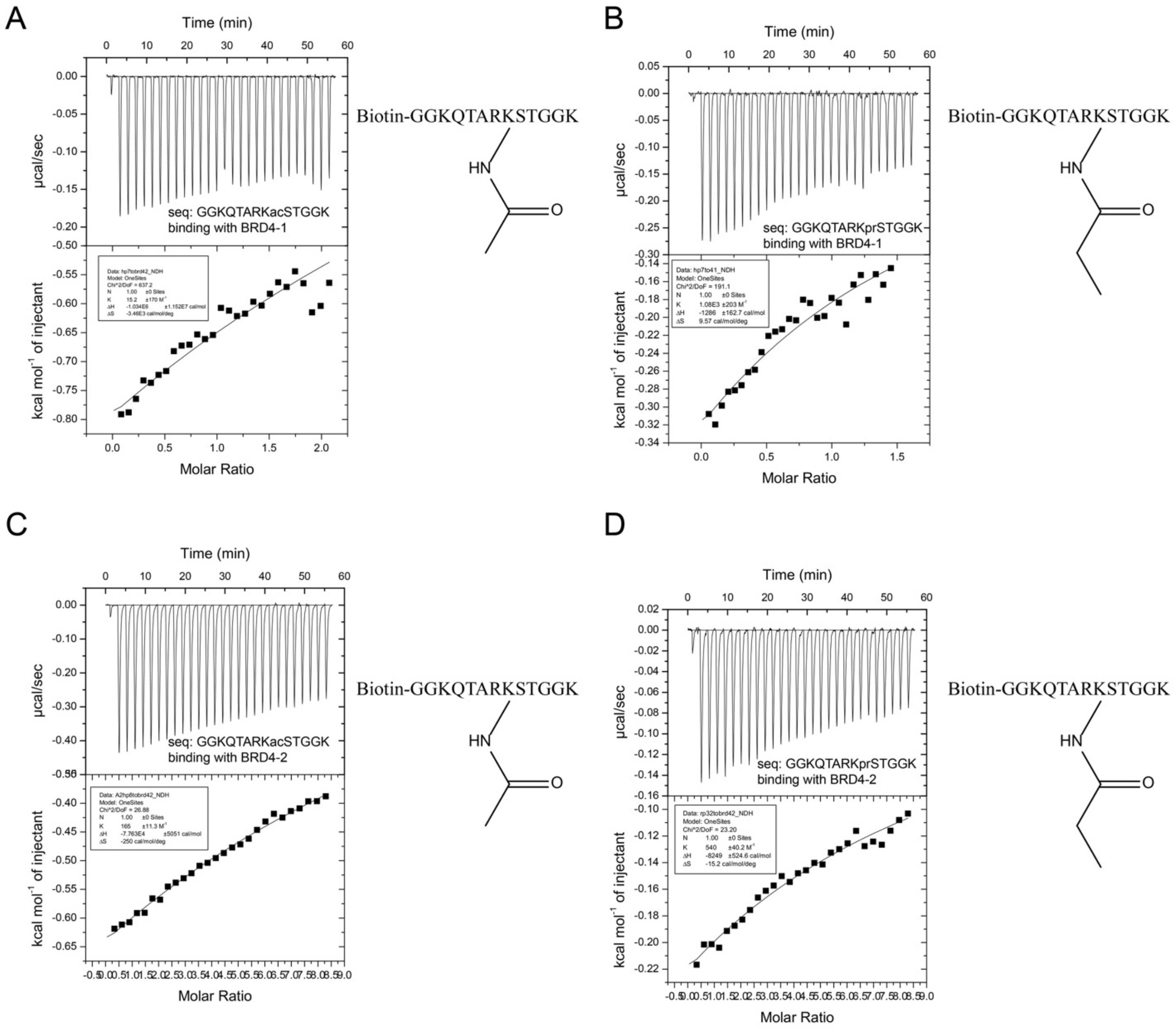

Figure S2 (A-B) ITC traces of acetylation histone H3k9 (Ac) and H3k9 (Pr) peptides binding to BRD4 (1). (C-D) ITC traces of acetylation histone H3k59(Ac) and H3k9 (Pr) peptides binding to BRD4 (2).
